# Effect of Perioperative Stroke on Survival After Carotid Intervention

**DOI:** 10.1177/15385744231207015

**Published:** 2023-10-18

**Authors:** Tommaso Cambiaghi, Alexander Mills, Samuel Leonard, Regina Husman, Syed T. Zaidi, Ezra Y. Koh, Kourosh Keyhani, S. Keisin Wang

**Affiliations:** 1 Division of Vascular Surgery, Department of Cardiothoracic and Vascular Surgery, 12339 McGovern Medical School at UTHealth Houston, Houston, TX, USA

**Keywords:** stroke, carotid intervention, endarterectomy, stenting, mortality

## Abstract

**Objectives:**

Perioperative stroke is the most dreaded complication of carotid artery interventions and can severely affect patients’ quality of life. This study evaluated the impact of this event on mortality for patients undergoing interventional treatment of carotid artery stenosis with three different modalities.

**Methods:**

Patients undergoing carotid revascularization at participating Memorial Hermann Health System facilities were captured from 2003-2022. These patients were treated with either carotid endarterectomy (CEA), transfemoral carotid stenting (TF-CAS), or transcarotid artery revascularization (TCAR). Perioperative outcomes, including stroke and mortality, as well as follow-up survival data at 6-month intervals, were analyzed and stratified per treatment modality.

**Results:**

Of the 1681 carotid revascularization patients identified, 992 underwent CEA (59.0%), 524 underwent TCAR (31.2%), and 165 underwent TF-CAS (9.8%). The incidence of stroke was 2.1% (CEA 2.1%, TCAR 1.7%, and TF-CAS 3.6%; *P* = .326). The perioperative (30-day) death rate was 2.1% (n = 36). The perioperative death rate was higher in patients who suffered from an intraoperative stroke than in those who did not (8.3% vs 1.9%, *P* = .007). Perioperative death was also different between CEA, TCAR, and TF-CAS for patients who had an intraoperative stroke (.0% vs 33.3% vs .0%, *P* = .05). TCAR patients were likely to be older (*P* < .001), have a higher body mass index (*P* < .001), and have diabetes mellitus (*P* < .001). Patients who suffered from an intraoperative stroke were more likely to have a symptomatic carotid lesion (58.3% vs 28.8%, *P* < .001). The TCAR group had a significantly lower survival at 6 months and 12 months when compared to the other two groups (64.9% vs 100% *P* = .007).

**Conclusion:**

Perioperative stroke during carotid interventions significantly impacts early patient survival with otherwise no apparent change in mid-term outcomes at 5 years. This difference appears to be even more significant in patients undergoing TCAR, possibly due to their baseline higher-risk profile and lower functional reserve.

## Introduction

Extracranial carotid atherosclerotic disease is a common etiology of thromboembolic ischemic stroke (15%-20%).^1^ In order to prevent emboli of carotid source, accepted interventional approaches have been developed consisting of the three established methods: carotid endarterectomy (CEA); transfemoral carotid stenting (TF-CAS); and transcarotid artery revascularization (TCAR).^2^ Despite these interventions being generally safe and effective, intraoperative complications do occur, with the most dreaded being intraoperative stroke and perioperative death.^3^ Although relatively uncommon, intraoperative stroke can severely affect a patient’s quality of life, and the incidence of this complication ranges from 1% to 5%.^4-7^

While TF-CAS has shown to have an increased risk of intraoperative stroke in clinical trials when compared to CEA, this approach is still indicated and preferred for select patients.^6,7^ TCAR, approved by the FDA in 2015, was introduced as a potential evolution of TF-CAS and has shown promising results. Nonetheless, very few studies have evaluated how intraoperative stroke impacts postoperative survival,^8,9^ and even less is known regarding how this effect differs across these three interventional modalities.

We sought to evaluate the impact of intraoperative stroke on short-term and mid-term mortality across CEA, TF-CAS, and TCAR at our institutions. These findings could help surgeons identify those patients at increased risk and guide clinical decision-making.

## Materials and Methods

### Patient Selection and Consent

This study was approved by the institutional review board at McGovern Medical School at UTHealth Houston and the affiliated Memorial Hermann Health System (HSC-MS-21-0772). Data handling and manuscript preparation was performed in accordance with the latest iteration of the Declaration of Helsinki.^10^ A retrospective review of a prospectively maintained carotid surgical intervention database was performed from 2003-2022. Patients in this database were entered from 7 hospitals across the Memorial Hermann Health System. There was no patient contact required for this study, so a waiver of informed consent was granted.

Patients were selected for carotid intervention based on the published SVS guidelines for extracranial carotid artery stenosis,^11^ and included both asymptomatic and symptomatic patients. Treatment modality was selected based on current recommendations and both clinical and anatomical factors at the discretion of the intervening physician.

The perioperative period was defined as 30 days from incision. When feasible, all patients were recommended medical therapy, consisting of statin and single antiplatelet therapy indefinitely. TF-CAS and TCAR patients were also recommended a second antiplatelet agent, unless already on oral anticoagulant therapy. In that case, oral anticoagulants were resumed after procedure completion.

### Outcomes and Statistical Techniques

Primary outcomes were stroke and death events in the perioperative and follow-up periods across all modalities. Outcomes of interest were compared by using Chi-square analysis, and short-term and mid-term mortality was estimated using time-to-event Kaplan Meier survival analysis. Descriptive characteristics were compared between the two groups by using Chi-square for binary variables and Mann-Whitney U for continuous variables. All statistical analysis was performed in RStudio (RStudio version 4.2.1, Boston, MA).

## Results

During the specified period, 1681 carotid revascularizations were captured in our database. Of these patients, 992 underwent CEA (59.0%), 524 underwent TCAR (31.2%), and 165 underwent TF-CAS (9.8%). Approximately one-third (29.4%) of our population exhibited a transient ischemic attack or stroke in the anterior or middle cerebral artery distribution in 180 days preceding the index carotid operation. The incidence of perioperative stroke was 2.1% (n = 36) across all modalities. Stroke rates between CEA, TCAR, and TF-CAS were 2.1% (n = 21), 1.7% (n = 9), and 3.6% (n = 6), respectively (*P* = .326).

The overall perioperative death rate among this cohort was 2.0%, and it was significantly higher in patients who suffered from a stroke than in those who did not (8.3% vs 1.9%, *P* = .007). Perioperative death was also different between CEA, TCAR, and TF-CAS for those patients who had a perioperative stroke (.0% vs 33.3% vs .0%, *P* = .05). Additionally, 35 of the 36 operative strokes were ipsilateral, while 1 one of them was contralateral (TCAR patient). Length of stay for those who did not have a perioperative stroke was lower than those who did have a stroke (1.0 days vs 7.0 days, *P* < .001).

### Demographics and Medications

A comparison of preoperative patient characteristics and their medications between stroke patients and no stroke patients can be seen in Table 1. Between these groups, preoperative characteristics were comparable, with diabetes mellitus being higher in the stroke group (52.8% vs 38.3%, *P* = .08). Stroke patients were also less likely to be on clopidogrel (55.6% vs 69.2%, *P* = .08).

**Table 1.** Demographics and Medications Between Intraoperative Stroke Groups. Continuous Variables are Described by Median (Interquartile Range).

Variable	Stroke (N = 36)	No stroke (N = 1645)	Total (N = 1681)	*P-*value
Demographics				
Female	47.2%	56.2%	56.0%	.284
Age (years)	72.3 (67.9-79.5)	72.1 (65.9-78.3)	72.1 (66.0-78.4)	.460
BMI (kg/m^2^)	28.5 (24.3-31.5)	27.5 (24.1-31.6)	27.5 (24.1-31.6)	.747
Coronary artery disease	38.9%	50.6%	50.5%	.164
Cardiac arrhythmia	19.4%	13.4%	13.5%	.290
Hypertension	97.2%	93.4%	93.5%	.359
Hyperlipidemia	77.8%	77.4%	77.4%	.962
Active smoker	22.2%	25.1%	25.0%	.697
COPD	16.7%	15.2%	15.2%	.806
Diabetes mellitus	52.8%	38.3%	38.6%	.077
Hemodialysis	2.8%	2.5%	2.5%	.916
Medications				
Aspirin	77.8%	84.0%	83.9%	.313
Clopidogrel	55.6%	69.2%	68.9%	.080
Other antiplatelet	2.8%	2.0%	2.0%	.727
Anticoagulation	13.9%	9.7%	9.8%	.399
Statins	86.1%	85.6%	85.6%	.934
B-blockers	38.9%	51.7%	51.4%	.130

BMI = body mass index; COPD = chronic obstructive pulmonary disease.

A comparison between the three interventional techniques can be seen in Table 2. Among the different surgical groups, TCAR patients were more likely to be older (*P* < .001), have a higher body mass index (*P* < .001), and have diabetes mellitus (*P* < .001). TF-CAS patients were more likely to have coronary artery disease (*P* = .02), hyperlipidemia (*P* < .001), and COPD (*P* < .001). For medications, TCAR patients were more likely to take aspirin (*P* < .001), clopidogrel (*P* < .001), other antiplatelets (*P* = .003), anticoagulation (*P* < .001), and statin (*P* < .001).

**Table 2.** Demographics and Medications Between Surgical Techniques. Continuous Variables are Described by Median (Interquartile Range).

	CEA (N = 992)	TCAR (N = 524)	TF-CAS (N = 165)	Total (N = 1681)	*P-*value
Demographics					
Female	57.8%	54.8%	49.7%	56.0%	.121
Age (years)	71.1 (65.2-77.0)	75.0 (68.6-80.8)	70.4 (62.7-77.3)	72.1 (66.0-78.4)	<.001
BMI (kg/m^2^)	27.5 (24.3-31.5)	28.0 (24.3-32.4)	25.8 (22.5-29.3)	27.5 (24.1-31.6)	<.001
Coronary artery disease	50.1%	47.9%	60.7%	50.4%	.016
Cardiac arrhythmia	10.9%	18.5%	13.5%	13.5%	<.001
Hypertension	94.8%	93.1%	87.1%	93.5%	.001
Hyperlipidemia	74.0%	81.9%	84.0%	77.4%	<.001
Active smoker	26.0%	22.3%	27.0%	25.0%	.234
COPD	12.4%	17.4%	25.8%	15.3%	<.001
Diabetes mellitus	36.2%	43.3%	38.0%	38.6%	.026
Hemodialysis	2.0%	3.2%	3.1%	2.5%	.309
Medications					
Aspirin	83.1%	88.7%	73.2%	83.9%	<.001
Clopidogrel	54.8%	93.9%	74.4%	69.0%	<.001
Other antiplatelet	1.0%	3.4%	3.0%	2.0%	.003
Anticoagulation	7.5%	13.4%	11.6%	9.7%	<.001
Statins	83.7%	92.6%	75.0%	85.6%	<.001
B-Blockers	51.3%	51.9%	50.0%	51.4%	.912

BMI = body mass index; COPD = chronic obstructive pulmonary disease.

### Anatomic and Intraoperative Characteristics

Table 3 displays anatomic and intraoperative characteristics comparing the stroke group to the no stroke group, and Table 4 shows this comparison between the different interventional techniques. Patients who suffered from a stroke were more likely to have a symptomatic carotid lesion (58.3% vs 28.8%, *P* < .001), and they were also likely to have a lower estimated blood loss (*P* < .001). Otherwise, anatomic and intraoperative variables were comparable between the groups. For the surgical techniques, TF-CAS patients were more likely to be treated for carotid restenosis (*P* < .001), had undergone prior neck dissection (*P* < .001), and prior neck radiation (*P* < .001). General anesthesia was used for TF-CAS only 9.8% of the time (*P* < .001). TCAR patients had a more prevalent administration of protamine (*P* < .001). CEA patients had a higher estimated blood loss (*P* < .001) and longer operative time (<.001).

**Table 3.** Anatomic and Intraoperative Characteristics of Both Intraoperative Stroke Groups. Continuous Variables are Described by Median (Interquartile Range).

Variable	Stroke (N = 36)	No stroke (N = 164**5**)	Total (N = 1681)	*P-*value
Anatomic				
Symptomatic lesion	58.3%	28.8%	29.4%	<.001
Time to revascularization (weeks)	.5 (.5-0.9	1.0 (.5-3.0)	.9 (.5-3.0)	.056
Bifurcation above C2	5.6%	2.2%	2.3%	.180
Restenosis	2.8%	6.0%	5.9%	.422
Previous neck dissection	5.6%	7.7%	7.6%	.636
Neck radiation	2.8%	2.5%	2.5%	.915
Spine immobility	.0%	.8%	.8%	.592
Contralateral occlusion	2.8%	2.1%	2.1%	.791
Intraoperative				
Technique				.326
CEA	58.3%	59.1%	59.1%	
TF-CAS	16.7%	9.6%	9.8%	
TCAR	25.0%	31.3%	31.1%	
General anesthesia	83.3%	85.2%	85.2%	.756
Protamine	63.6%	77.1%	76.8%	.070
Estimated blood loss (cc)	40.0 (13.8-200.0)	50.0 (10.0-100.0)	50.0 (10.0-100.0)	<.001
Operative time (mins)	82.0 (67.0-101.0)	73.0 (56.0-96.0)	73.0 (56.0-96.0)	.103

CEA = carotid endarterectomy; TF-CAS = transfemoral carotid artery stent; TCAR = transcarotid revascularization.

**Table 4.** Anatomic and Intraoperative Characteristics of the Different Surgical Techniques. Continuous Variables are Described by Median (Interquartile Range).

	CEA (N = 992)	TCAR (N = 524)	TF-CAS (N = 165)	Total (N = 1681)	*P-*value
Anatomic					
Symptomatic lesion	27.6%	32.6%	29.7%	29.4%	.128
Time to revascularization (weeks)	1.0 (.6-4.0)	.6 (.5-3.3)	.9 (.5-2.0)	.9 (.5-3.0)	.051
Bifurcation above C2	1.6%	3.4%	2.4%	2.3%	.076
Restenosis	1.4%	8.0%	26.1%	5.9%	<.001
Previous neck dissection	1.6%	9.4%	38.2%	7.6%	<.001
Neck radiation	.9%	2.3%	12.7%	2.5%	<.001
Spine immobility	.5%	1.5%	.0%	.8%	.048
Contralateral occlusion	1.7%	2.3%	4.2%	2.1%	.112
Intraoperative					
General anesthesia	91.7%	96.2%	9.8%	85.1%	<.001
protamine	73.5%	89.1%	57.1%	76.8%	<.001
Estimated blood loss (cc)	50.0 (10.0-100.0)	20.0 (10.0-50.0)	10.0 (10.0-10.0)	50 (10.0-100.0)	<.001
Operative time (mins)	90.0 (74.0-113.0)	56.0 (46.0-68.0)	63.5 (48.8-82.5)	73.0 (56.0-96.0)	<.001

CEA = carotid endarterectomy; TF-CAS = transfemoral carotid artery stent; TCAR = transcarotid revascularization.

### Survival Analyses

**Figure 1.**
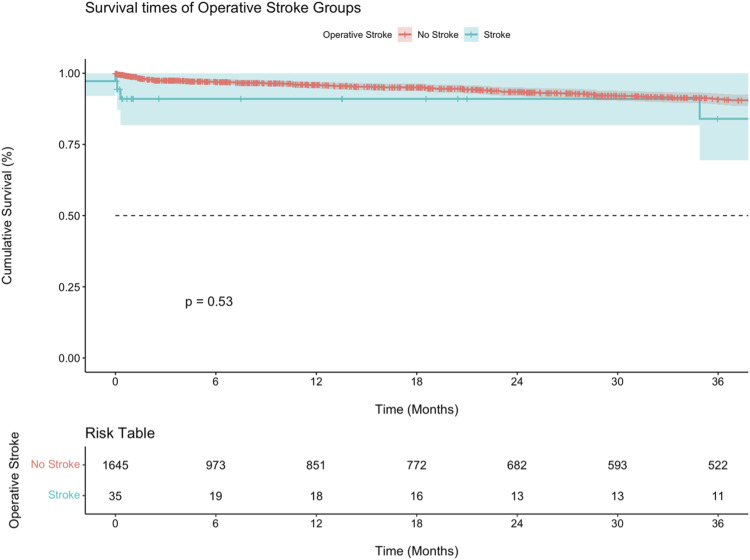
Kaplan-Meier curve comparing mortality between patients who suffered from an intraoperative stroke vs those who did not. All surgical modalities are included.

**Figure 2.**
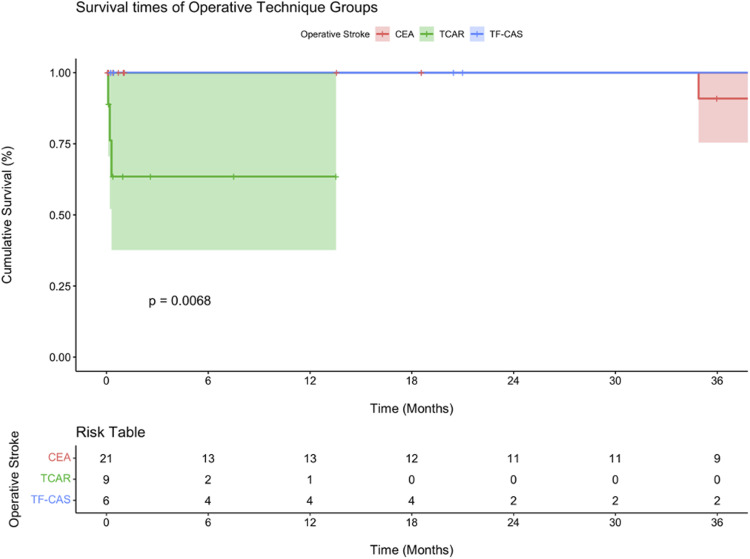
Kaplan-Meier curve comparing mortality between CEA vs TF-CAS vs TCAR in those patients who suffered from an intraoperative stroke.

Kaplan-Meier analysis was performed to compare survival between perioperative stroke and non-stroke patients (Figure 1), as well as to compare survival across different treatment modalities in patients who had a stroke. (Figure 2) Overall, the two groups did not show a statistically significant survival difference (*P* = .53). At 30 days, the median survival was noticeably lower in the stroke group (90.9% vs 99%), but this difference grew smaller over time with a 5-year median survival of 83.9%, compared to 86.0% for the non-stroke group (not shown in Figure 2). The TCAR group had a significantly lower estimated survival at 6 and 12 months compared to the other two groups (*P* = .007). The median survival at these time periods were 64.9% as compared to 100.0% for the CEA and TF-CAS groups.

## Discussion

In this study, we evaluated 1681 consecutive patients who underwent carotid surgical interventions at 7 hospitals within a unified health system. The purpose was to evaluate the effect of intraoperative stroke on perioperative death and mid-term survival across three interventional treatment modalities—CEA, TCAR, and TF-CAS. Overall, the rate of perioperative stroke and operative death was low and consistent with contemporary literature.^12,13^

As expected, 30-day mortality increased in patients who suffered from a perioperative stroke event. Additionally, patients undergoing TCAR who suffered from a stroke were at increased risk of perioperative mortality when compared to the other surgical cohorts. It is unclear if this is a statistical anomaly or if the severity of stroke with TCAR is increased, given the potentially higher likelihood of watershed compared to embolic events.^14,15^ The difference in mortality decreased over time and minimized at 5 years postprocedure. Stroke events after carotid interventions significantly impact early patient survival, and this difference appears to be even more significant in patients undergoing TCAR. This result could also be related to the selection bias intrinsic to the experience with TCAR in the early period, when this treatment modality was FDA-approved only for high-risk by anatomy or physiology patients. Consequently, these patients, with lower baseline physiological reserve, suffer more significantly from the impact of perioperative stroke and consequent disability.

A randomized trial comparing TCAR to CEA and TF-CAS would help clarify the real impact of perioperative stroke on overall survival by eliminating selection bias. Furthermore, as TCAR became approved for standard-risk patients, we expect this difference in survival to become less prominent because of more homogenous patient populations. Unfortunately, such a study is not likely to occur soon due to the exorbitant numbers required to adequately power it and the associated costs.

Last, the stroke rate across the whole study population was reasonably low and, despite not reaching statistical significance, TCAR appeared to have the lowest stroke rate (1.7% vs 2.1% for CEA and 3.6% for TF-CAS). This is consistent with what has been reported from national databases, such as the Vascular Quality Initiative TCAR Surveillance Project.^16^ Besides procedure-specific factors influencing this outcome, we recorded more prevalent use of best optimal medical therapy in the TCAR group, with more consistent use of statin and DAPT therapy (as required by TCAR protocol) when compared to the other two groups.

There are several limitations in this study to keep in mind when evaluating the results. First, as a retrospective analysis, variables are often missing or misinterpreted. Also, loss of follow-up over the course of time affects the accuracy and validity of the Kaplan Meier tests. Heterogeneity between hospitals, patient populations, and practice patterns are all present. Last, selection bias between the three revascularization modalities limit our ability to compare results directly and, therefore, we generally avoid that in this analysis.

## Conclusion

This study shows that perioperative stroke after carotid revascularization decreases early but not midterm survival at 5 years. This difference is even more significant in patients undergoing TCAR compared to TF-CAS and CEA.
